# Initial adaptation among university student: The case of the social sciences

**DOI:** 10.1371/journal.pone.0294440

**Published:** 2023-11-13

**Authors:** Franciele Corti, Juan Llanes, Inmaculada Dorio Alcaraz, Montserrat Freixa Niella

**Affiliations:** 1 Department of Education and Humanities, Universitat Abat Oliba CEU, CEU Universities, Barcelona, Catalonia, Spain; 2 Department of Research and Diagnostics in Education, Institut de Recerca en Educació, Universitat de Barcelona, Barcelona, Catalonia, Spain; 3 Department of Research and Diagnostics in Education, Universitat de Barcelona, Barcelona, Catalonia, Spain; University of Huelva: Universidad de Huelva, SPAIN

## Abstract

Students’ academic and social adaptation is linked to factors such as their initial study motivations, the atmosphere of the academic environment and their perception of academic wellbeing. This paper analyses the initial adaptation of first-degree students in their first semester through a quantitative ex post facto study with a descriptive-exploratory approach, using a questionnaire as the information-gathering instrument. Findings shed light on the concept of initial adaptation itself (through the creation of an index) and validate the study of the construct through three factors: initial motivation, the academic environment and perception of academic wellbeing. Also, the influence on initial adaptation of the academic and social context of the degree course taken is demonstrated:, as the constitutive features of the degree contribute information predicting how students will integrate into the institution. Therefore, it is suggested that institutional means and actions should be designed and put in place in accordance with measurements that indicate how students function best in a specific context (the bachelor’s degree), in order to boost motivation and the perception of academic wellbeing.

## Introduction

Every year students with varying social and academic profiles come together in first-year university classrooms, mixing conventional or traditional profiles with less conventional ones [1, 2]. The former arrive to their bachelors’degrees directly from sixth forms, while the latter increasingly gain university access through advanced vocational training (the Spanish equivalent of BTEC), which has reversed the social and academic profile of the majority of students on some social and juridical sciences degrees [1, 3]. Other new arrivals are mature students of over 25 and 45, who have a different social-academic profile with its own particularities, and lastly, other under-represented profiles such as students with disabilities [3]. This heterogeneity of social and academic pathways of university access contributes to the multi-factor, dynamic and temporary nature of contemporary students’ transition to university life, attesting to the interest of a study of the initial adaptation process through which students seek to settle into the university and persist in it until they have achieved their objectives.

It is in their first year that university students establish the relationships with the environment they need to respond to the various situations of adaptation they face: fitting into a peer group, building a support network, comparing and adjusting their prior expectations to their current experience, and fulfilling their academic tasks successfully. This process culminates when they obtain their first academic results and show that they are capable of persisting [4–6].

In this context, academic and social adaptation becomes a complex construct due to its interactive, multi-factor and dynamic nature [4, 7]. It is interactive because it is the outcome of multiple, continuous interactions between the student and the environment; it is multi-factor because neither contextual factors nor personal ones can be understood independently in the process of adaptation [8]; and it is dynamic because, in turn, the student-environment relationship causes successive changes in both that can facilitate (or not) academic and social adjustment.

In interaction with the university context, students’ entry variables are highly important, i.e., the *academic and social background* of the student who is in the process of adaption. A meta-analysis performed by Dupont et al. (2015) [9] concluded that previous academic performance, university entrance grades and students’ social origins explained both university success and failure or drop-out. Thus the student’s social origin becomes a predominant variable, particularly associated with the profiles of non-conventional students, who tend to persist less or lag behind in their academic performance [10]. Various studies have confirmed these findings [11], revealing the influence of the means of university access on performance and persistence among non-conventional students. Nevertheless, although social origin mediated by good-quality school and family experience results in university success, some studies [12] reveal that this relationship varies according to the university institutions (faculties) that students enrol in, thereby both suggesting and verifying the influence of the educational context.

In the light of the above, it was considered pertinent to investigate whether students’ social and academic access profiles determined the outcome of initial adaptation and whether this process was affected by the degree chosen.

In other contexts, such as the USA and in Portugal, Lent et al. (2005, 2009) [13, 14] have related initial adaptation to the analysis of students’ academic satisfaction on the basis of the social cognitive theory of the degree (SCCT) [13–17]. Their purpose was to analyse and understand how the multi-faceted process of initial adaptation developed during the first year at university. In this perspective, perceived initial adaptation, defined as students’ belief in their ability to adjust their socio-academic entry profile to the university context, was linked to academic satisfaction, perceived initial motivation and perceived academic stress. *Academic satisfaction* was defined as the degree of wellbeing students felt in academic situations at university. *Perceived initial motivation* referred to the degree of fulfilment of the student’s socio-academic expectations when participating in the university context. Lastly, *perceived academic stress* was defined as students’ ability to deal positively with adverse situations, both academic and social, that might affect their expectations of results and their progress towards their goals, and in consequence their self-efficacy [14, 16].

Adopting an interactionist approach, which links students to the context, Tinto (2012) [18] studied the adaptation process through three factors: motivation, the academic environment and perception of academic wellbeing.

The decision to undertake university studies involves a process of research and selection, in which students prioritize motives that can respond to personal values and aspirations, or to external factors, such as family expectations or social stereotypes [19]. Motivation, therefore, is composed of extrinsic elements related to economic advancement or social status, or of intrinsic motives associated with learning and professional life, expressed in the academic field in terms of the enjoyment of learning and the desire for achievement linked to successful personal and professional development [20]. Intrinsic motivation, moreover, can be connected with contextual factors (teachers, peers, classroom climate, academic stimulation, etc.) that reinforce students’ progress through their university careers [21], or with institutional factors that strengthen the support offered by the university administration, such as the secretary’s office, counselling and guidance services and the study plan itself [22, 23].

*Motivation* is a factor that is seen to predict success in students’ first-year transition [24], together with belief in self-efficacy. According to Robbins et al. (2004) [25] and Lent et al. (2005) [13], people tend to develop and persist in those activities in which they feel themselves to be effective and can obtain positive results. Bailey and Phillips’ (2016) [26] findings show that students with intrinsic motivation have a stronger feeling of wellbeing, are more satisfied and attain better academic results. Thus throughout their first year, students gradually incorporate into their lifestyles the features of the university environment that enable them to feel increasingly autonomous and competent in their integration into the context and active participation in it. Students have to address personal, social and institutional situations that test their capacity to take on new responsibilities and thereby persist throughout their academic and professional careers [27].

The *academic environment* has been confirmed as another decisive factor in students’ adaptation processes. During their first year, this factor sets in motion personal, social and academic mechanisms for integrating and adapting to the new context [5, 27]. Students pass from a familiar social and academic setting to a new one whose organisation and functioning they are unfamiliar with and do not yet know how to interpret. Thus they need a pre-existing support network, both academic and social, to help and guide them through this first year [9]. The social and academic environment of the faculty where students take their first degree is shaped by an organisational and functional structure that permeates the whole system, ranging from institutional interactions that help or hinder the adaptation process [5] to what Tinto (2012, 2017) [6, 18] calls the classroom climate, the classroom being the space where students find the support that they need to help them understand and interpret the institutional and social keys to adapting and succeeding in the new context.

Lastly, turning to the *perception of academic wellbeing*, studies of first-time students’ experience of the university both in our context [28] and internationally [12, 29] have shown that satisfaction in the first experiences of university is an excellent predictor of academic and social adaptation. From this perspective, perception of academic wellbeing manifests when students interact positively with their surroundings, feel that they belong there and, also, effectively use strategies to respond to the academic demands of the new context [27, 30].

## Materials and methods

In this study we approached initial adaptation (academic and social) through the prism of motivation, the academic environment and the perception of academic wellbeing in students’ experience in the first semester of their first year on the degrees of Pedagogy, Business Administration and Management (BAM) and Early Childhood Education at the University of Barcelona (UB).

The study was organised around four questions, which also comprised the specific objectives of the study:

1. With the objective of identifying the factors shaping the construct of initial adaptation: *How are the items of the construct of initial adaptation articulated in the Spanish context*?
2. With the objective of determining the factors shaping university students’ access profiles: *What are current university students’ entry-level variables*?
3. With the objective of investigating the factors making for heterogeneity in university student profiles, and to ascertain how this heterogeneity interferes with their initial adaptation: *What are the relationships between the items in the construct of initial adaptation and students’ entry-level variables*?
4. With the aim of determining the personal and adaptive variables that were key to explaining degree choice: *Does the context of the specific degree determine students’ processes of initial adaptation*?

In order to address these questions and to fulfil the study’s general and specific objectives, a quantitative ex post facto study was designed, adopting a descriptive-exploratory approach with a questionnaire as the information-gathering instrument.

### Sample

The population analysed was the whole set of students on the degrees of Pedagogy, BAM and Early Childhood Education at the Faculty of Social and Juridical Sciences, University of Barcelona. These three degrees were chosen because they embody different conditions and because of their representativeness within the field of social sciences, their importance at the institutional level and the ease of access to their data, since their heads of study were members of the research team.

The administration of the questionnaire in the 2010–11 academic year coincided with the coming into force of Royal Decree 1892/2008, which gave unlimited university access to graduates of advanced vocational training courses, thus increasing the number of students entering the university through this pathway. Although at first sight it may seem that the data are not up-to-date, in fact since the application of Royal Decree 1892/2008, there have been no changes to the relevant degree progammes. In view of this context and in view of the imminent changes stemming from the new Royal Decree 822/2021 and the Universities Law (LOSU), our data contribute to discussion of on the future study plans of the degree programmes analysed.

The study was performed between February and March of the 2010–11 academic year. The questionnaire was administered *in situ* in the classrooms of all the groups of students enrolled in the first year. Access to the classrooms for this purpose was arranged with the respective degree heads of studies. Likewise, students’ authorisation was sought for the use of their personal and academic data, ensuring confidentiality.

All the students who attended class on that particular day, then, responded to the questionnaire. Obligatory modules were chosen in order to ensure the presence of the whole study population. Although all students in the population were invited to participate, the sample was finally composed of those who voluntarily agreed to participate in the study; therefore the sampling technique was non-probabilistic by accessibility.

The sample totalled 1,764 students, of which 953 answered the questionnaire, 54.02% of the overall population, giving a sample error of 2.15 and confidence level of 95% (see Table 1).

**Table 1:** Population and sample of the study.

Degree	Population	Sample
**BAM**	1,290	651
**Pedagogy**	233	140
**Early Childhood Education**	241	162
**TOTAL**	1,764	953

The demographic data of the sample were as follows: 35.8% were male and 64.2% female; the mean age was 21.34, with a standard deviation of 3.473; 37.1% were working as well as studying; 85.2% were taking their degree of first choice; 61.4% had come to the university straight from sixth form, 26.5% from vocational training, 10.1% from other university degrees, and 1.9% as mature students above the age of 25 or 45.

### Data-gathering instrument

With the aim of evaluating students’ experiences of their academic transition processes at a key time (after the first trimester assessment period), data were collected using the First Trimester Assessment Questionnaire (FTAQ), adapted by the UB Academic and Occupational Transitions Research Group (TRALS) from the Portuguese version of a scale by Lent et al. (2005) [13, 14].

All eight items of the five-point scale were analysed (Conbrach’s α: 0.736). In addition, a item on motivation item was included by the TRALS research team, and a further five-point item on academic stress also with five rating points was retrieved from the original academic adjustment scale by Lent et al. (2005) [13]. Conbrach’s α showed a reliability of 0.809 for the ten-item scale.

Demographic variables obtained from the university’s enrolment database were also analysed, namely: degree, timetable, mode of enrolment, work, sex, age, type of residence, scholarship, means of access, admission grades, choice of degree, father’s studies, mother’s studies, father’s occupation, mother’s occupation.

The study adhered to the social science standards of the ethical committee at the University of Barcelona (Spain). Informed consent was obtained from all subjects involved. All participants were ensured anonymity and informed of the reasons behind the study and how their data would be used.

### Procedure and data analysis

Data was collected on the degrees of Pedagogy and BAM from the RDI project “Student persistence and drop-out during the first year of Social Science degrees at university: foundations for improving retention”, and in the degree of Early Childhood Education from the thesis “Transition to University. The Degree in Early Childhood Education”.

The authors of this article have focused their attention on the analysis of part of the questionnaire, specifically on the constructs: of adaptation, motivation and well-being, scrutinizing the entry variables in order to determine how the transition to university is made (specifically on the three degrees studied) and how the entry profile marks (or does not mark) possible differences.

An inductive process was followed in presenting the findings, which were adjusted on the basis of the data obtained and their significance. Below, in order to clarify the sequence, we explain why the findings are presented on the basis of the tests chosen and not others.

One specific objective of the article was to examine the constructs analysed in the theoretical framework (for the Spanish sample). To this end, a factor analysis was carried out based on the core concepts of the article. The purpose of this was to attempt to make advances on previous studies on the concept of intial adaptation, which in our case underpinned the construction of the questionnaire itself. Thus, after carrying out the factor analysis, it was considered to be important to present it first in order to provide an appropriate frame for the rest of the findings.

Regarding the profile of the sample, it was initially planned to present a description of all the variables analyzed, but since the information obtained would lengthen the article and as this was not a specific determining objective, it was decided to highlight only those variables that characterised the differential access profiles. After performing a number of tests to investigate the significance of the data, it was observed that the determining variable with most explanatory power for the profile was that of the admission grade. For this reason, it was this variable that was finally chosen as the root node of the decision tree.

To detect possible differences between the three constructs analyzed in the factor analysis and all the input variables (regardless of whether or not they were significant in explaining the sample profile) it was considered important to analyse all of them in detail. For this reason a micro-analytical approach to the data was chosen, using non-parametric tests for two or more independent samples. This enabled us to go into greater depth, with the purpose of unearthing new factors to take into consideration in improving future degree courses.

In the micro-analytical analysis, it was again the degree variable of the degree that marked the difference in relation to the rest of the variables when it was linked it to the concepts of the adaptation construct. This variable had also affected the roadmap in the analysis of the in-put variables. This led the team to perform a regression analysis to ascertain how this variable (a determinant variable) explained the prediction for the initial adaptation. The model reported a 48% justification of variance, thus yielding an adequate fit for its prediction.

The data analysis was performed using the SPSS v.23 program and the AMOS v.18 complement.

In order to analyse the internal validity of the construct and thus respond to the first research question, firstly an exploratory factor analysis (EFA) was carried out, followed by a confirmatory factor analysis (CFA), with the aim of identifying the best fit for the model [31].

The EFA was conducted with the method of principal component analysis with Varimax rotation, since there was no dominant factor. Extraction of the main components was chosen, since the purpose of the study was to “identify the number and composition of the components necessary to summarise the scores of a large set of variables […]. This method explains the maximum percentage of variance found in each item on the basis of the smallest number of components summarising this information” [25, p. 1153]. The factor retention method adopted took into account the percentage of variance explained (59.76% of total variance). The test’s reliability was confirmed by the Kaiser-Meyer-Olkin value (KMO = 0.795, sig. = 0.000) and a Bartlett test (sig. = 0.000). The results of the EFA coincided with the theoretical model.

The CFA was performed using estimates of maximum likelihood (MLR). Different theoretical models from other contexts were initially considered ([30] in the Portuguese context and [13, 32] in the North American context) alongside the model emerging from the EFA (in accordance with the theoretical model set out in the introduction). Finally, it was chosen to confirm that the model emerging from the EFA provided adequate fit to the data. This was analysed through the combined use of robust fit measures and incremental fit, when, due to the size of the sample, the value of the chi square test (χ2 = 255.814, p<0.001) indicated lack of fit of the model [33]. The root mean square error of approximation (RMSEA = 0.086 [0.076, 0.096]), between 0.10 and 0.05, showed an acceptable fit for the model [34]. The comparative fit index (CFI = 0.909) was considered acceptable for values above 0.9 [35]. The other measures of incremental fit were also good (IFI = 0.910; NFI = 0.898), as they were above or very close to 0.9 [36]. The combined results indicated a suitable fit to the data of the model emerging from the EFA.

To answer the second research question on students’ entry profile (background), a decision tree was created. In the segmentation test, the type of degree was taken as a dependent variable, and the entry profile variables included in the questionnaire as independent. CHAID (Chi-square Automatic Interaction Detection) is a method of classification that builds decision trees using chi-square statistics to determine how variables best combine to explain the outcome. Only the following variables were included in the classification model: grouped admission marks, gender, means of access, working while studying and age. A total of 16 nodes were obtained with a depth of three levels and a risk estimate value of 0.232. The resulting model classified and predicted correctly 76.8% of all cases, with the highest percentage of correctness among students taking BAM, as opposed to Pedagogy.

To answer the third research question a microanalysis of the items making up each factor was performed, crossing each item with the entry variables. When a Kolmogorov-Smirnov test was performed on the sample it was observed that the bilateral asymptotic significance “p” equalled 0.000 in all cases (p<0.05). This meant that the variables did not follow normal law and that, in consequence, parametric tests could not be applied. For this reason non-parametric microanalytical tests were carried out.

Lastly, the fourth research question was investigated by performing an analysis to determine which variables from the entry profile and the adaptation scale were decisive in explaining the degree chosen. To this end a stepwise multiple linear regression model was applied [19].

## Results

### How are the items in the construct of initial adaptation articulated in the Spanish context?

First, in order to understand how the items in the construct of initial adaptation were grouped in our context, an EFA was performed. This identified three independent factors (see Table 2), in which all the items obtained saturation values of over 0.05 (i.e. above the minimum recommended value [37]) in their corresponding factor. Bearing in mind both the theory underlying the study and the data emerging from the statistical analysis, a study was made of the components that led to the choice of name for each factor, i.e.: factor 1: motivation; factor 2: academic environment; and factor 3: perception of academic wellbeing.

**Table 2:** Matrix of rotated components resulting from the EFA.

Matrix of rotated components			
	Component		
	1	2	3
I’m satisfied with my chosen degree	.588		
I’m comfortable with the educational atmosphere		.824	
I’m enjoying studying here	.522		
I’m satisfied with my life as a student			.564
I like the level of academic stimulation in the classes		.742	
I’m satisfied with the modules	.739		
I like what I’m learning in class	.805		
My current academic adaptation is …			.503
My current motivation to study is …	.626		
I feel capable of coping with academic difficulties			.870

^a^ Extraction method: principal components analysis. Rotation method: Varimax with Kaiser normalisation.

^b^ The rotation converged in four iterations.

The first factor explained 24.297% of total variance and represented students’ motivation to study their chosen degree. The second factor explained 18.755% of variance and encompassed the elements of the academic environment (educational atmosphere). Lastly, the third factor explained 16.706% of variance and grouped together the elements associated with the students’ perception of academic wellbeing.

**Fig 1:**
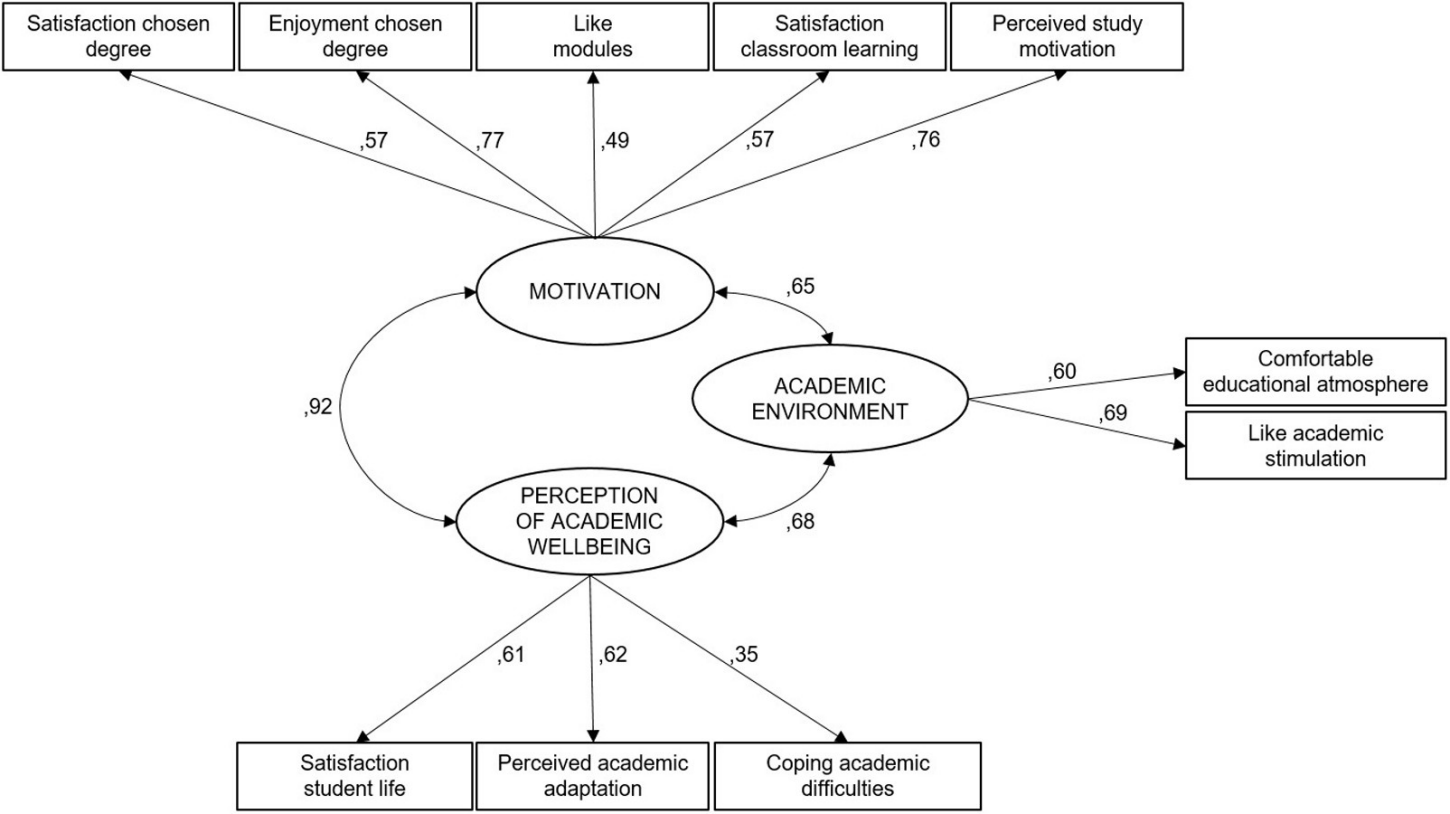
CFA of the construct of academic adaptation.

The CFA was used to test the validity of the internal structure, which enabled us to confirm to what extent the data ratified the factor model of the scale emerging from the EFA. Fig 1 shows the result of this analysis, in which the saturation values of the items associated with each factor can be observed.

### What are current university students’ entry variables?

**Fig 2:**
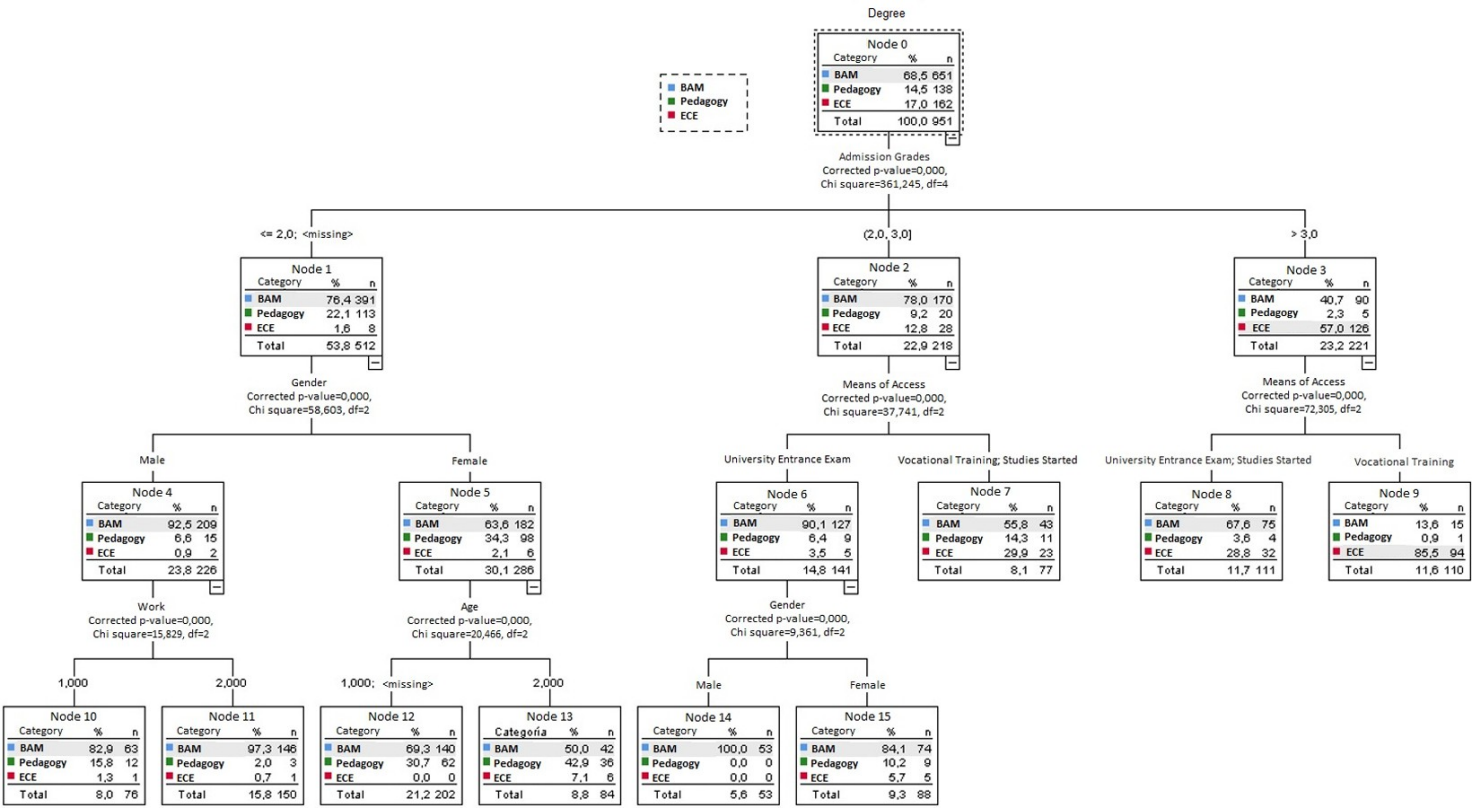
Classification of students according to degree using a CHAID decision tree statistical test.

The tree diagram (Fig 2) represents the classification of the 951 participants into 16 nodes. This analysis addressed the second research question. It can be observed in the first level that the university admission grade was a differentiating element in defining what type of students enrolled on the degree and under what conditions (Chi² = 361.245 gl = 4 sig .000). The admission grade was lower for BAM students and higher for Early Childhood Education (57.1% needed less than 9, while 77.8% needed more than 10).

The following significant classification was divided only by two elements: gender (512 participants) and means of access (439 participants). In gender, mostly females in Pedagogy and males in Business Administration with lower grades acceded (72.3% and 81.2% respectively with less than 9). In means of access, the students with the highest grades came from vocational training in the case of Early Childhood Education and from university entrance exams or other degrees in BAM (66.5% with more than 10 and 59% with less than 10 respectively).

The third and last level of the classification tree shows gender, work (whether or not the student was working and studying concurrently) and the hours worked as important factors in the entry profile. On the BAM degree, all those arriving at university with medium grades were male. In BAM and Pedagogy a high percentage of males gaining access with lower grades were also working (60.3% and 52% respectively). The women arriving with lower grades were younger in BAM (72%), while there was a high percentage of mature students in Pedagogy (49.6%). In BAM, most men gaining access with a low grade did not work (63%).

In short, the variable with the highest predictive power was the degree, followed by the admission grades. The whole set of students in node 4 was that which best predicted and classified the tree itself, with a total of 286 participants in this box. This node was defined by mainly young women with low admission marks. Node 11 was differentiated from this node by the nuance that this type of student was especially prominent in Pedagogy, followed by BAM.

Other variables were analysed, yielding differentiating results. Although these were not important in building the decision tree, they were statistically significant. The sample mostly comprised young students living with their families of origin (65%; Chi² = 57.466 gl = 4 sig 0.000). Students with grants were mainly found in Early Childhood Education (42.6%; Chi² = 11.042 gl = 2 sig 0.004) (although only 1/3 of the sample had been awarded any kind of economic aid). Students’ fathers mostly had primary-school qualifications and worked in low-qualified jobs (78.8%; Chi² = 71.895 gl = 6 sig 0.000), and for their mothers this was exactly the same in 80% of cases (Chi² = 23.111 gl = 6 sig 0.001). Lastly, a high percentage of female students were not working (39.6%).

### What was the relationship between the items in the construct of initial adaptation and students’ entry variables?

Below the results of each of the items in the construct of initial adaptation are presented in relation to students’ entry variables, focusing on the three factors describing the construct (motivation, academic environment, and perception of academic wellbeing). An item-by-item microanalysis was performed to probe in greater depth the differences between students arriving at the university and to analyse how their entry variables influenced their initial adaptation.

Table 3 shows the items in the first factor (motivation) with their significant differences, giving the measures for each independent variable, on a scale of one to five points.

**Table 3:** Items for the factor of motivation and entry variables.

^1 2^			Degree chosen	Enjoyment of studies	Modules	Classroom learning	Motivation
**Degree**		Pedagogy (138)	4.30	4.09	3.28	3.96	3.79
		BAM (651)	4.05	3.57	3.25	3.67	3.32
		Early Childhood Education (162)	4.53	3.42	2.77	3.65	3.35
	*Test Statistics*	*Chi-square*	*62.629*	*50.220*	*37.74*	*19.615*	*33.807*
		*df*	*gl 2*	*gl. 2*	*gl. 2*	*gl. 2*	*gl. 2*
		*Asymp. Sig*	*(.000)*	*(.000)*	*(.000)*	*(.000)*	*(.000)*
**Timetable**		Morning (650)		3.59			3.33
		Afternoon (268)		3.71			3.51
		Both (35)		3.51			3.63
	*Test Statistics*	*Chi-square*		*6.060*			*10.810*
		*df*		*gl. 2*			*gl. 2*
		*Asymp. Sig*		*(.048)*			*(.004)*
**Working**		Yes (352)			3.07		
		No (598)			3.24		
	*Test Statistics*	*U de Mann-Whitney*			*92.510*		
		*df*			*-3*,*163*		
		*Asymp. Sig*			*(.002)*		
**Type of residence**		Family home (823)					3.36
		Hotel or residence (49)					3.45
		Own flat (39)					3.90
		Other (42)					3.50
	*Test Statistics*	*Chi-square*					*13.775*
		*df*					*gl. 4*
		*Asymp. Sig*					*(.008)*
**Means of access**		University entrance exam (585)	4.09	3.65	3.24	3.69	
		Second degree (97)	4.30	3.80	2.80	3.80	
		Vocational training (253)	4.25	3.47	2.98	3.65	
		Older than 25 or 45 (18)	4.33	3.70	2.83	4.17	
	*Test Statistics*	*Chi-square*	*18.637*	*11.101*	*1*.,*549*	*11.772*	
		*df*	*gl. 4*	*gl. 4*	*gl. 4*	*gl. 4*	
		*Asymp. Sig*	*(.001)*	*(.025)*	*(.001)*	*(.019)*	
**Admission grades**		-8 (241)	4.10	3.63	3.17	3.69	3.33
		-9 (223)	4.08	3.71	3.30	3.79	3.42
		-10 (210)	4.11	3.55	3.14	3.61	3.39
		-11 (180)	4.37	3.49	3.02	3.67	3.33
		+11 (41)	4.47	4.07	3.45	4.05	4.04
	*Test Statistics*	*Chi-square*	*29.119*	*16.637*	*13.166*	*18.384*	*23.028*
		*df*	*gl. 5*	*gl. 5*	*gl. 5*	*gl. 5*	*gl. 5*
		*Asymp. Sig*	*(.000)*	*(.005)*	*(.022)*	*(.003)*	*(.000)*
**Father’s education**		Primary school (372)					3.38
		Secondary school (210)					3.50
		Higher education (219)					3.32
		None (32)					3.66
	*Test Statistics*	*Chi-square*					*8.150*
		*df*					*gl. 3*
		*Asymp. Sig*					*(.043)*
**Mother’s occupation**		Qualified (531)		3.69			
		Unqualified (16)		3.19			
		Not specified (404)		3.54			
	*Test Statistics*	*Chi-square*		*10.44*			
		*df*		*gl. 2*			
		*Asymp. Sig*		*(.005)*			
**Gender**		Male (340)	4.08				
		Female (611)	4.22				
	*Test Statistics*	*U de Mann-Whitney*	*94235.5*				
		*df*	*-2.595*				
		*Asymp. Sig*	*(.009)*				
**Age**		-20- (504)	4.15		3.23	3.67	
		+20 (334)	4.26		3.05	3.77	
	*Test Statistics*	*U de Mann-Whitney*	*76757.5*		*75212.5*	*76689.5*	
		*df*	*-2.365*		*-2.579*	*-2.398*	
		*Asymp. Sig*	*(.018)*		*(.010)*	*(.016)*	
**Grant**		Yes (313)	4.24				
		No (640)	4.13				
	*Test Statistics*	*U de Mann-Whitney*	*91982*				
		*df*	*-2.241*				
		*Asymp. Sig*	*(.025)*				

^a^ Kruskal-Wallis test. Chi-square (for more than two independent samples). Differences were corroborated by applying the effect size from Dunn’s test for independent samples.

^b^ Mann-Whitney U. Value Z (two independent samples). Differences were corroborated by ap-plying the effect size from the Dunn-Bonferroni test for independent samples.

The variable of the degree involved differences for all the items in this factor. In terms of degree course chosen, students on the Early Childhood Education degree were the most satisfied, while those taking BAM were the least satisfied. The rest of the variables were more determining for Pedagogy students. In enjoyment of their courses and modules, Early Childhood Education students indicated less satisfaction. BAM and Early Childhood Education students had low medium scores in motivation and classroom learning.

Regarding the class timetables, students taking the degree only in the afternoons were those who enjoyed their courses most, and those dividing their time between the mornings and afternoons enjoyed them the least, while the latter group had higher motivation.

Whether the student worked or not influenced satisfaction with the modules taken, as those not working expressing greater satisfaction in their choices.

Motivation to study was lower among students living with their families and higher among those who lived in their own flats.

Regarding the means of university access, those who were beginning a second degree or who had entered university as mature students over 25 or 45 were the most satisfied and most enjoyed their courses, unlike those coming from sixth forms and vocational training, while those who stated that they were most satisfied with their modules were those who had come from sixth forms, the least satisfied with modules being those on their second degree or over 25/45. Mature students enjoyed their classroom learning most, whereas those coming from vocational training enjoyed this least.

Analysing admission grades, those students who had gained access to the university with higher marks were more satisfied with all the items analysed.

Children of fathers without educational qualifications stated that they were most motivated to study, while the least motivated were those whose fathers had university degrees. Children of mothers who were qualified workers enjoyed their studies more than children of unqualified or unclassified mothers.

Women students, mature students and those in receipt of grants were more satisfied with the degrees chosen. The youngest were those who were happiest with the modules studied, although the oldest stated that they were more satisfied with their classroom learning.

Table 4 shows the second factor (academic environment) with the significant differences and measures for each independent variable, on a scale of one to five points.

**Table 4:** Items of the academic environment factor and entry variables.

^1 2^			Educational atmosphere	Academic stimulation
**Degree**		Pedagogy (138)	4.17	3.48
		BAM (651)	3.71	2.78
		Early Childhood Education (162)	3.81	3.06
	*Test Statistics*	*Chi-square*	*40.087*	*62.246*
		*df*	*gl. 2*	*gl. 2*
		*Asymp. Sig*	*(.000)*	*(.000)*
**Type of residence**		Family home (823)	3.82	
		Hotel or residence (49)	3.37	
		Own flat (39)	3.54	
		Other (42)	3.80	
	*Test Statistics*	*Chi-square*	*16.506*	
		*df*	*gl. 4*	
		*Asymp. Sig*	*(.002)*	
**Degree choice**		First (812)	3.79	2.91
		Second (74)	3.62	2.81
		Third (31)	4	3.13
		Fourth or more (36)	4.14	3.42
	*Test Statistics*	*Chi-square*	*10.912*	*12.302*
		*df*	*gl. 3*	*gl. 3*
		*Asymp. Sig*	*(.012)*	*(.006)*
**Mother’s occupation**		Qualified (531)		3.01
		Unqualified (16)		3
		Not specified (404)		2.83
	*Test Statistics*	*Chi-square*		*7.340*
		*df*		*gl. 2*
		*Asymp. Sig*		*(.025)*
**Gender**		Male (340)	3.79	2.74
		Female (611)	3.85	3.04
	*Test Statistics*	*U de Mann-Whitney*	*92378.5*	*86609*
		*df*	*-3.131*	*-4.330*
		*Asymp. Sig*	*(.002)*	*(.000)*

^a^ Kruskal-Wallis test. Chi-square (for more than two independent samples). Differences were corroborated by applying the effect size from Dunn’s test for independent samples.

^b^ Mann-Whitney U. Value Z (two independent samples). Differences were corroborated by ap-plying the effect size from the Dunn-Bonferroni test for independent samples.

Students on the Pedagogy degree were more satisfied with the educational atmosphere and the level of academic stimulation in class, in comparison to those on BAM.

With regard to their place of residence, students living at home or with a member of their family were more satisfied with the educational atmosphere of the university than those living in student flats or halls of residence.

The students whose degrees were their third or fourth choices were those who were happiest with the educational atmosphere and academic stimulation in class, while those who had chosen their degree as their second option were the least satisfied with these items.

As for stimulation in class, this was higher for children of mothers in qualified work than those who had not specified their mothers’ occupations.

Turning to gender, female students were more satisfied with the educational atmosphere and academic stimulation.

The items in the third factor (perception of academic wellbeing) are shown in Table 5, measured on a scale of one to five points, which summarises the significant differences and the measures for each independent variable.

**Table 5:** Items from the factor of perception of academic wellbeing and entry variables.

^1 2^			My student life	Academic adaptation	Coping with difficulties
**Degree**		Pedagogy (138)	4.20	4	3.46
		BAM (651)	3.87	3.56	2.99
		Early Childhood Education (162)	3.52	3.76	2.39
	*Test Statistics*	*Chi-square*	*47.598*	*45.978*	*87.325*
		*df*	*gl. 2*	*gl. 2*	*gl. 2*
		*Asymp. Sig*	*(.000)*	*(.000)*	*(.000)*
**Working**		Yes (352)	3.76		
		No (598)	3.91		
	*Test Statistics*	*U de Mann-Whitney*	*95835.5*		
		*df*	*-2.539*		
		*Asymp. Sig*	*(.011)*		
**Means of access**		University entrance exam (585)	3.95	3.67	3.10
		Second degree (97)	3.80	3.80	3.20
		Vocational training (253)	3.66	3.56	2.54
		Older than 25 or 45 (18)	3.89	3.33	2.72
	*Test Statistics*	*Chi-square*	*18.943*	*15.522*	*67.380*
		*df*	*gl. 4*	*gl. 4*	*gl. 4*
		*Asymp. Sig*	*(.001)*	*(.004)*	*(.000)*
**Admission grades**		-8 (241)		3.57	2.95
		-9 (223)		3.63	3.14
		-10 (210)		3.68	3.09
		-11 (180)		3.74	2.62
		+11 (41)		4.12	2.80
	*Test Statistics*	*Chi-square*		*22.160*	*30.971*
		*df*		*gl. 5*	*gl. 5*
		*Asymp. Sig*		*(.000)*	*(.000)*
**Choice of degree**		First (812)			2.96
		Second (74)			2.68
		Third (31)			3.13
		Fourth or more (36)			3.33
	*Test Statistics*	*Chi-square*			*12.411*
		*df*			*gl. 3*
		*Asymp. Sig*			*(.006)*
**Mother’s education**		Primary (413)			2.86
		Secondary (192)			3.04
		Higher (210)			3.11
		None (33)			2.82
	*Test Statistics*	*Chi-square*			*8.687*
		*df*			*gl. 3*
		*Asymp. Sig*			*(.034)*
**Mother’s occupation**		Qualified (531)	3.90	3.72	3.05
		Unqualified (16)	3.25	3.56	2.06
		Not specified (404)	3.83	3.58	2.87
	*Test Statistics*	*Chi-square*	*7.747*	*8.862*	*21.861*
		*df*	*gl. 2*	*gl. 2*	*gl. 2*
		*Asymp. Sig*	*(.021)*	*(.012)*	*(.000)*
**Father’s occupation**		Qualified (621)		3.71	3.02
		Unqualified (35)		3.34	2.71
		Not specified (295)		3.58	2.85
	*Test Statistics*	*Chi-square*		*12.232*	*9.213*
		*df*		*gl. 2*	*gl. 2*
		*Asymp. Sig*		*(.002)*	*(.010)*
**Gender**		Male (340)		3.55	3.08
		Female (611)		3.72	2.88
	*Test Statistics*	*U de Mann-Whitney*		*96940.5*	*91982*
		*df*		*-2.817*	*-2.915*
		*Asymp. Sig*		*(.005)*	*(.004)*
**Age**		-20- (504)	3.94	3.71	3.04
		+20 (334)	3.70	3.59	2.76
	*Test Statistics*	*U de Mann-Whitney*	*72922*	*77546*	*70334*
		*df*	*-3.592*	*-2.062*	*-4.119*
		*Asymp. Sig*	*(.000)*	*(.039)*	*(.000)*

^a^ Kruskal-Wallis test. Chi-square (for more than two independent samples). Differences were corroborated by applying the effect size from Dunn’s test for independent samples.

^b^ Mann-Whitney U. Value Z (two independent samples). Differences were corroborated by ap-plying the effect size from the Dunn-Bonferroni test for independent samples.

Pedagogy degree students felt more satisfied with their lives as students than those taking Early Childhood Education. Academic adaptation was more positive in Pedagogy and less so in BAM. Pedagogy students also felt more prepared to cope with difficulties, while those taking Early Childhood Education felt less so. Students who were not working were more satisfied with their student lives.

Students gaining access to university through higher vocational training stated that they were not satisfied with the lives as students, while the most satisfied in this respect were students over 25 or 45. The latter were those who saw themselves as least adapted, while those who were beginning a second degree scored highest in both their academic adaptation and their ability to cope with difficulties. The group that felt least able to face academic difficulties were those coming from vocational training.

Students who had gained access to university with higher grades (over 10) had adapted better than those who had entered with lower grades (over 8). Nevertheless, students with intermediate marks (from 8 to 10) felt less able to tackle academic difficulties than those with lower marks.

The group of students whose degrees were their third or fourth choices stated that they were more able to face difficulties compared with those whose degrees were their second choice.

Students whose mothers had university qualifications felt more able to cope with academic difficulties than those whose mothers had no qualifications. Students with fathers in qualified work stated that they felt more academically adapted and with greater ability to tackle difficulties than those whose fathers were in unqualified occupations. Students with mothers holding qualified jobs enjoyed their lives as students more than those with mothers in unqualified work, and also stated that they had adapted better to the university and coped better with academic difficulties.

Female students were more satisfied with their academic adaptation than male. The latter, however, felt more capable of coping with difficulties. The youngest felt more satisfied with their lives as students and more able to adapt to the university and their studies and to cope with difficulties.

### Did the context of the university degree determine students’ processes of initial adaptation?

Regarding the degree chosen, the regression analysis showed that the predictor variables were: the means of access, gender, the admission grades, working or not, satisfaction with the chosen degree and modules, the order of degree choice, the level of academic stimulation in class, the class timetable, the fathers’ educational background and the ability to cope with difficulties in the courses chosen (see Table 6). The coefficient of determination of the predictive model for the degree was: r = 0.703, R² corrected = 0.494, and R² adjusted = 0.485, which suggests that the chosen variables predicted 48% of the variability of the total score related to the degree taken.

**Table 6:** Linear regression model for the degree variable.

	Unstandardised coefficients		Standardised coefficients		
Model	B	Std. Error	Beta	t	Sig.
Constant	1.354	.216		6.270	.000
Means of access	.186	.022	.259	8.50	.000
Gender	-.472	.052	-.269	-9.120	.000
Admission grades	.175	.020	.266	8.844	.000
Satisfaction with chosen degree	.206	.032	.191	6.472	.000
Satisfaction with modules	-.156	.028	-.168	-4.516	.000
Working	-.203	.049	-.121	-4.121	.000
Order of choice	.173	.039	.130	4.424	.000
Level of academic stimulation	.074	.025	.089	2.960	.003
Timetable	-.120	.043	-.081	-2.819	.005
Father’s education	-.062	.025	-.071	-2.474	.014
Coping with difficulties	-.056	.025	-.067	-2.253	.025

Regarding gender, Pedagogy and Early Childhood Education were predominantly female (88.4% and 97.5% respectively), while in BAM the percentage of males and females was roughly equal (49.2% and 50.8%). The model suggested that the majority of students still devoted most of their time to their studies, although this statistic differed according to the degree: in BAM 70.7% were full-time students, in Pedagogy 53.6% and in Early Childhood Education 39.5%.

Students of BAM and Pedagogy mostly came to their degree via sixth form (72.5% and 63.8% respectively), while the majority of Early Childhood Education students came from vocational training courses (70.4%). Although 40.6% of students taking Pedagogy had an afternoon timetable, generally the students’ classes were in the morning. Most were studying on their first-choice degrees and had university access with grades very close to those required.

Parents’ educational backgrounds were still mainly primary school for all three degrees, although in Pedagogy 33.1% of students had parents with secondary qualifications and in BAM 32.7% had parents with university degrees.

Most students from all three degrees expressed a moderate ability to cope with academic difficulties, with those in Pedagogy scoring highest in this respect. Regarding the level of academic stimulation, it was again students of Pedagogy who stated that they were the most satisfied, followed by Early Childhood Education students, and lastly BAM, who indicated low stimulation. The students who were most satisfied with their choice of degree were those of Early Childhood Education, followed by Pedagogy, with BAM last. In the area of the modules studied, the most satisfied were those studying BAM, followed by Pedagogy and lastly Early Childhood Education.

## Discussion

Two major topics of discussion emerge from the results of this study. The first centres on the construct of initial adaptation itself and its relationship with the entry variables shaping students’ access profiles. This first topic corresponds to our three first research questions. The second focuses on the influence of the specific degree on the process of initial adaptation, and corresponds to the fourth research question.

In response to our second research question, in general terms, our results confirmed the findings of prior studies [5, 12, 18, 38–40]. Despite educational policies intended to open up access to higher education, *students’ academic and social background* still determines their initial adaptation and process of transition. Even today, students with conventional entry profiles adapt to the university environment more easily and make a more successful transition than those with non-traditional profiles, who are more susceptible to dropping out [41].

Turning to the first question, the main topic of this study, we found that, even using the questionnaire adapted from the initial adaptation model in Lent et al. (2005) [13], the factor analysis showed that inductively the data fitted best with Tinto’s interactionist approach [18, 42, 43]. Also, it was confirmed that the items in the construct of initial adaptation in Spain were grouped into three factors: motivation, perception of wellbeing and the academic environment; thereby demonstrating that the adaptation process is not generalisable and that the entry variables determine the weight of each factor.

With regard to the factor of *motivation*, the students coming to university with higher admission marks were those who felt most motivated to study their chosen degrees and enjoyed their classroom learning most. They were academically well-trained students who easily connected with the university system and had resources for effective recognition, interpretation and action when taking on the academic and social challenges of university study [44].

Turning to the socio-economic status of students’ families, those whose mothers were in skilled work were more academically motivated. In the case of the father, however, students’ initial motivation was related to fathers with no educational qualifications, contradicting the findings of studies from other countries [4, 9]. These results indicate the importance of the family environment, which can both motivate the younger generation to take a degree and provide essential support [45].

We should also note the importance of the personal maturity of students who were older (mature students and those taking second degrees) and more independent (with their own accommodation [4]). Thus motivation acquired associations with personal and professional self-realisation, factors driving students towards successful transitions [19].

The in-depth analysis of the factor underpinning the construct of initial adaptation, the *perception of academic wellbeing*, strengthened its links with previous academic performance (i.e., the university entrance mark [11, 29, 46]), with social and family environments in which the university context is familiar (parents with higher education [3, 7, 30]), and with full-time study (whether the student worked or not) as entrance variables that facilitated initial adaptation [26, 27]. These variables coincided with the conventional student entry profile [1, 2, 47].

With regard to the *academic environment* factor, studies such as those by Tinto (2012, 2017) [6, 18] and De Clercq et al. (2018) [12] suggest that some variables, such as socio-economic origin for example, can be nuanced by the degree courses chosen, thereby showing the importance of the educational context in academic success. Thus the focus for a successful transition is the context and not the student, and the university should advance beyond the reproduction of prevailing social norms and certain forms of cultural and social capital [48]. The academic culture of the degree may thus become important; and this brings us to the second major topic of discussion.

In this regard, then, our micro-level analysis of the factors and variables in each degree revealed differences between the entry variables and the different factor items. This prompted the question of whether the type of degree attracted a particular type of student, or a particular type of student enrolled in a certain type of degree. Whatever the focus of the question and answer, however, there was no doubt that there were differences in student profiles according to the degrees.

The Pedagogy degree mostly comprised working females with low admission grades who were not taking their first-choice course. Despite this, the degree and their classroom learning motivated them, they felt comfortable with the educational environment and the level of stimulation in class, and they were satisfied with their student life and their ability to overcome difficulties. Thus it would seem that both the degree and the environment offered them a stimulating educational experience, thereby overcoming the difficulties in initial adaptation associated with their entry variables [4, 10, 12].

In contrast, Business Administration students who had chosen the degree as their first option, and whose family support had been vital to their university access, were not happy with their course. This was the least motivated group and that which perceived the least help from educational actors. These findings suggest that the degree can determine initial adaptation [30]. In the analysis of this course (which has a high student-teacher ratio), the classroom context did not constitute a favourable environment for integration into the university [44, 48].

Students of Early Childhood Education, mostly female, came from alternative academic routes (such as advanced vocational training), and for the majority the degree was their first choice. Thus they were satisfied with their course, but not with the specific modules studied. Neither were they happy with their student life, and they also had difficulties in taking on academic challenges. Hence there was a discrepancy between their expectations and the real situation they encountered. Their university education differed from the training received on preparatory courses, which had a high degree of professionalisation [49]. These conclusions should help university managers optimise the factors that can contribute to a successful transition [6, 13].

## Conclusions

This study sheds light on the construct of initial adaptation. On the one hand, this construct was validated on the basis of the three factors enabling its clarification: initial motivation, academic environment and perception of academic well-being. This concurs with prior studies such as those by Lent et al. (2005; 2009) [13, 14] and Almeida et al. (2019) [30], although not fully coinciding with their classifications. The exploratory factor analysis and the literature review enabled us to adjust the factors making up initial adaptation to our context, adapting its definition to the situation studied.

Furthermore, the direct influence on initial adaptation of the culture of the degree itself was demonstrated; in other words, the degree and its academic and social context bear the greatest influence on the initial integration process. The constitutive features of the course itself predicted students’ inclusion in the institution to a larger extent than the rest of the variables studied, including the entry variables (indicators of students’ personal contexts) associated with the heterogeneity of the access profiles.

As in all research, we should consider, at the end of the process, the limitations of the study itself. In terms of methodology, although the sampling error was acceptable, the data should be interpreted with caution, since only the students attending class responded and those absent may have afforded a different view of the degrees analyzed. In future research, it would be interesting to carry out comparative analyses between cohorts of students and to investigate the differences in the construct itself. Furthermore, although the exploratory and confirmatory analyses were satisfactory, it would also be advisable to expand the sample to improve the fit of the model and to carry out comparative studies with other countries in order to understand the theory and practice of higher education in general and to obtain global results that could promote equity policies on an international level (which could also be seen as a limitation of this study).

It should also be taken into account that the data, although current, are from a cohort prior to the covid-19 pandemic, and that the situation in universities has been transformed due to this [46]. Not only the educational methods and practices but also the way students experience and understand the university and how they relate to each other and their teachers have changed. These factors will surely condition the construct under analysis. Our findings, however, are still valid for tracing the paths to be explored and the routes to follow.

This study was based on the analysis of academic satisfaction made by Lent et al. (2005) [13], in which the authors included new factors in order to examine the construct of initial adaptation in greater depth [50]. Although the factors analyzed yielded information on adaptation, in future research it would be advisable to follow the same line as Tinto (2012, 2017) [6, 18], including items from the factors of academic environment and perception of academic well-being [27].

Also, our data indicate the need for more research into non-conventional profiles in order to specify entry variables in greater depth and thus enable the creation of policies adapted to the specific reasons that lead to more successful transitions to university, in terms of persistence and graduation. In this line of study, it seems that maturity and student independence or autonomy are facilitating variables for initial integration; these variables, therefore, should be studied further in future projects [49, 51].

It is necessary to continue analyzing the attention and orientation facilities provided by faculties for students at the outset of their university careers. We would recommend enhancing students’ experience of university via their motivations and interests [52]; that is, taking into account their expectations and other possible variables motivating them to enroll in the degree. Students’ initial adaptation improves when their institutional culture is inclusive and participatory [21], and therefore a microanalytical approach to each degree is fundamental. Work should continue along this line, according to which, for example, tutorial action plans should be adapted to the new situations and contexts, as we are in a time of change that makes it necessary to reconsider not only the degree course itself, but also all complementary actions designed to contribute to the better functioning of programmes, faculties and universities.

## Supporting information

S1 Data (SAV)
